# A Field Collection of Indigenous Grapevines as a Valuable Repository for Applied Research

**DOI:** 10.3390/plants11192563

**Published:** 2022-09-28

**Authors:** Shani Shecori, Mafatlal M. Kher, Kamal Tyagi, Larry Lerno, Yishai Netzer, Amnon Lichter, Susan E. Ebeler, Elyashiv Drori

**Affiliations:** 1 Chemical Engineering Department, Ariel University, Ariel 40700, Israel; shanishamaka1@gmail.com (S.S.); mafatlalmkher@gmail.com (M.M.K.); ynetzer@gmail.com (Y.N.); 2 Eastern Regional R&D Center, Ariel 40700, Israel; 3 Horticulture Section, SIPS, Cornell University, Ithaca, NY 14853, USA; tyagi.kamal6673@gmail.com; 4 Department of Viticulture and Enology, University of California, Davis, CA 95616, USA; lalerno@ucdavis.edu (L.L.); seebeler@ucdavis.edu (S.E.E.); 5 Department of Postharvest Science, The Volcani Institute, Rishon LeZion 7528809, Israel; vtlicht@agri.gov.il

**Keywords:** Ex situ conservation, diversity, drought stress, field germplasm, *Vitis*, wine

## Abstract

The grapevine is an economically important plant, with a historical connection to the development of human culture. Currently, over 6000 accessions are known as individual grapevine varieties, some of which are important to national heritage, valuable for current viticultural practices, and as genetic resources to maintain plasticity under changing climatic conditions, environmental sustainability, and market demands. Recently, the diversity of cultivated grapevines has declined significantly, due to the increased focus of global wine industries on a few major cultivars. Moreover, due to biotic and abiotic stresses, the wild *V. vinifera* germplasm’s genetic diversity has declined, with some varieties on the verge of extinction. *Vitis* germplasm conservation can be achieved via either in situ (e.g., protected areas) or Ex situ (e.g., field collections, seed banks, and tissue culture collections) methods. This study aims to highlight the importance of *Vitis* field bank collections. We demonstrate the research done in the Israeli indigenous *Vitis vinifera* collection. The multi-layer analysis of the varieties enabled the identification of drought stress-resistant varieties, and suggested a mechanism for this resistance through noting the dramatic phenological differences in foliage development between resistant and sensitive varieties. In addition, we show a general characterization of the varieties via major grape characteristics, including bunch and berry shape, as well as their possible utilization based on their aromatic and phenolic profiles.

## 1. Introduction

Grapevine (*Vitis vinifera* L.) is an economically important fruit species worldwide, and has a historical connection with the development of human culture. Grapevine comprises cultivated (*V. vinifera* subsp*. sativa*) and wild forms (*V. vinifera* subsp*. sylvestris*). More than 6000 accessions are recorded as individual varieties [1]. Some are rare and have only a few unique vines that are important to national heritage, and are valuable as resources for cultivation and breeding. In the last few decades, the cultivated grapevine has experienced a drastic reduction in diversity due to the increased focus of the global wine industry on a few major cultivars [2]. Moreover, the loss of natural habitat is adversely affecting the genetic diversity of the wild *V. vinifera* species, with some populations on the verge of extinction [3]. Hence, immediate action to conserve indigenous grapevine germplasms is required.

*Vitis* germplasm conservation can be achieved either in situ (e.g., protected areas) or Ex situ (e.g., botanic gardens, seed banks, and tissue culture collections). In situ conservation refers to preserving a species in its native environment, and preserving and recovering viable populations in their natural habitat. However, anthropogenic activities and abiotic or biotic stress may lead to the extinction of the germplasm. On the other hand, ex situ conservation refers to preserving germplasm outside their native habitat. The methods include using slow growth tissue culture, cryopreservation, and seed banks; or preservation of the whole plant in a botanical garden or in a field gene bank; and via greenhouse cultivation of plant material. Using a slow growth tissue culture under in vitro conditions on minimal nutrient medium (restricted growth approach) ensures minimum maintenance costs by retarding growth rates [4]. However, this approach has several limitations, including the need for technical expertise, plant loss due to contamination of cultures, high labor costs, and the possibility of obtaining somaclonal variations [5,6]. Cryopreservation (at −196 °C) in liquid nitrogen also provides an opportunity for the long-term preservation of *Vitis* germplasm, which can then be used as a backup for field collections for important indigenous cultivars [7,8]. However, exposing cells to extremely low temperatures can result in freezing injury; hence, cells must be carefully handled and prepared before being frozen in liquid nitrogen. There is also the possibility of a loss of accessions occurring during the revival, either due to contamination or low recovery [9], especially for some specific *Vitis* varieties [10]. Seed bank storage (at −20 °C) is also utilized for the ex situ conservation of trees, although the germinating progeny will show segregating genotypes. However, plants with recalcitrant (desiccation-sensitive) and intermediate (relatively desiccation-tolerant) seeds cannot stand desiccation conditions and cold storage without losing viability; only orthodox seeds (desiccation-tolerant) are highly suited to this procedure. Moreover, conservation via storing seeds is suitable only for wild grapevines, and not for cultivated ones. Therefore, field gene bank collections are important for such species.

In field gene bank collections, tree species are maintained and multiplied indefinitely, using seeds or vegetative propagation; this emphasizes the importance of having a long-term plan for the sustainability of such collections. However, maintenance of such collections is costly (limited funding, cultivation land) [11], and extensive maintenance actions (labor, irrigation, disease and pest management) are required. Moreover, these collections are vulnerable to climate change, biotic and abiotic stresses, and sometimes natural disasters [2,12,13,14,15,16,17,18,19,20].

The emergence of intense competition in the international wine market has encouraged the idea of recovering high-quality ancient and indigenous accessions. As a result, a large number of indigenous accessions have been collected from wild and old vineyards, and maintained in the field collections of grapevine-growing countries, including Israel [21], the USA [22], Spain [23,24], Italy [25], and many other countries [23,26]. For example, the United States Department of Agriculture—Agricultural Research Service collections of wild grapevines at the Plant Genetic Resource Unit (Geneva, Switzerland; New York, NY, USA) and the National Clonal Germplasm Repository (Davis, CA, USA) maintain around 5000 accessions as a field collection [22]. The FEM grape germplasm collection (ITA362) at San Michele all’Adige, Italy, maintains 2273 accessions [25]. The IFAPA research center “Rancho de la Merced” (Jerez, Spain) hosts 930 accessions [23], and the “El Encín” Vine Varieties Collection, Spain, has 966 indigenous accessions [24]. Finally, the Vitis International Variety Catalogue (VIVC) is a database of ~23,000 cultivars, breeding lines, and *Vitis* species, that provides a comprehensive overview of most international grapevine collections [23,26].

The grapevine is one of the most sensitive cultivated plants; it is very responsive to its surrounding environment. Different varieties may have a wide range of responses to specific stressors; thus, maintaining a wide range of cultivars in a field collection using a uniform growth protocol is challenging [27]. As mentioned above, grapevine field collections also require large spaces, are labor-intensive, expensive to maintain [11], and are exposed to natural disasters, climate change, and infection by pathogens [2,12,13,14,15,16]. Therefore, the maintenance of field grapevine collections is challenging.

In spite of these complications, the importance of maintaining grapevine field collections is growing in light of current and forecasted climate changes; the sustainability of current commercial grapevine varieties is under greater pressure as a result of global warming. New, resistant varieties will be greatly needed [28,29,30], and it is anticipated that the diversity found in germplasm collections will become increasingly valuable for future breeding programs (e.g., for molecular markers resistant to biotic and abiotic stresses). Genomics-assisted breeding will reduce the time and costs that are required to breed new cultivars that possess desirable traits [31]. The methodical collection of data from a field collection can enhance our understanding of phenological variations, and of individual responses to various biotic and abiotic factors—for instance, in terms of cold hardiness [32,33], blooming time [34], drought stress [35,36], as well as important commercial traits such as wine characteristics [27,35]. Due to the adaptability of such varieties in local microclimatic conditions, these varieties can provide rootstocks that are resistant to the salt [37] and drought stresses that are associated with climate change [38].

Israel is characterized by a dramatic climatic gradient, from the northern moderate-temperature and humidity of the Golan Heights, to the southern hot and dry Negev [27]. Here, we describe the advantages of maintaining a unique field indigenous collection in Ariel, Israel. This collection includes 96 accessions that were collected in Israel during the last decade [39,40], representing one of the most ancient grape populations in the world, and one that is suspected of having been domesticated from local *Vitis sylvestris* populations [21]. Furthermore, individual grapevines in this collection were collected from very harsh ecosystems, including the Mediterranean Sea shore, the Negev desert, and dry mountainous areas; such grapevines represent a unique opportunity to study abiotic stress resistances. This collection was used to gain a better understanding of the genomic structure of the domesticated and wild grape populations in the south Levant [21,41,42]. This study will argue the benefits of maintaining this unique in-field collection in order to study drought resistance, phenotypic and phenological variations, and grape aroma profiles of previously uncharacterized grape varieties.

## 2. Results

In the following paragraphs, we will demonstrate the benefits of maintaining a grapevine germplasm field collection by showing results retrieved from the collection on various applied research aspects.

### 2.1. Drought Tolerance Characterization of Varieties in the Collection

**Figure 1:**
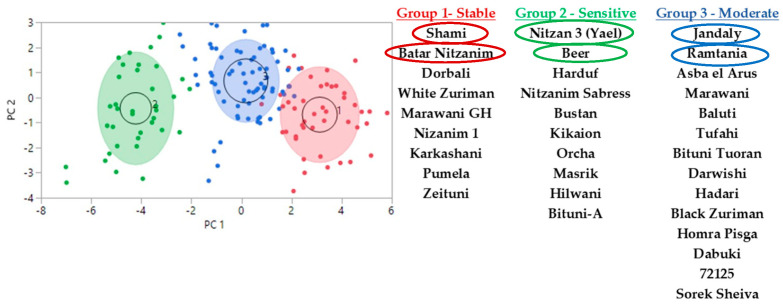
K-means cluster analysis and clustering of the 33 tested varieties into strategic groups. At five time points, the varieties were clustered by their physiological parameters during an extended drought stress experiment. The physiological parameters measured were carbon fixation (photosynthesis) (An), stomata conductance (g_s_), and stem water potential. Group 1—stable varieties, showing improved parameters at the late stages of the experiment, marked red. Group 2—sensitive varieties that reacted dramatically to the extended drought period, marked green. Group 3—varieties with a moderate response, marked blue. Each dot represents data for a specific vine, four vines per variety, for a total of 132 vines. Circles are drawn over varieties that were selected for emphasis in the following figures (Figure 2 and Figure 3).

**Figure 2:**
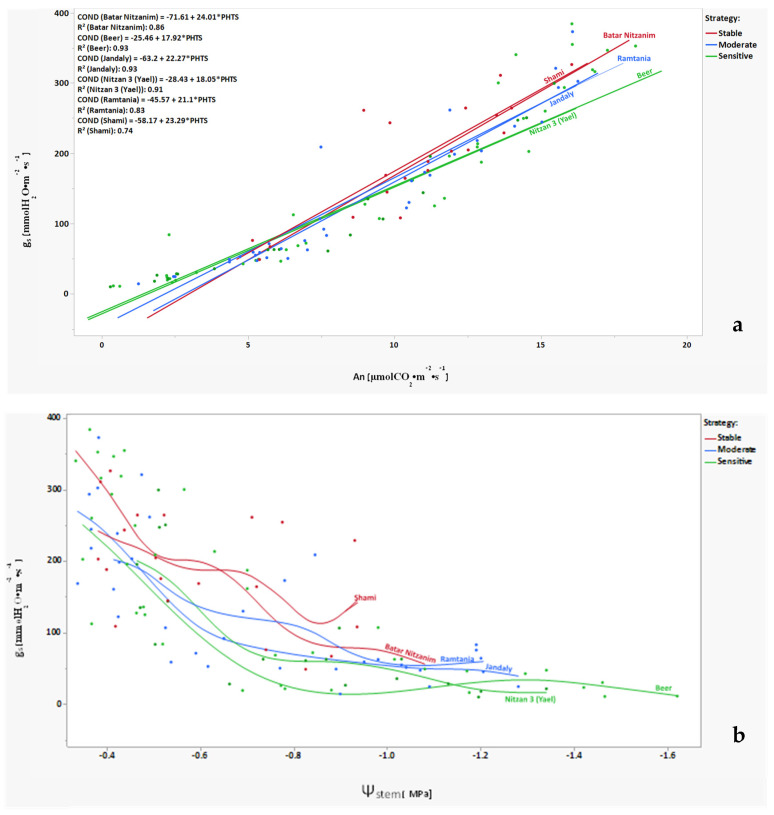
Correlations between physiological parameters during drought stress experiment. (**a**) Correlation between stomata conductance (g_s_) and photosynthesis (An). Two varieties represent each response strategy. (**b**) Correlation between stomata conductance (g_s_) and stem water potential (Ψ_stem_). Two varieties represent each response strategy. Each dot represents data for a specific vine at a specific time point during the drought experiment, four vines per variety.

This study presents the physiological data retrieved from 33 varieties (132 vines), measured at five time points during a drought stress experiment of 10 weeks. Using k-means analysis (k-means clustering divides the varieties by their observation data into k groups, so that data points in the same cluster are comparable, and that data points in other clusters are further apart) to classify the individual vines into groups [using the physiological information measured for each vine at all five time points—stem water potential (SWP), photosynthesis, and stomata conductance], we show that the vines, represented by dots, are divided into three groups that are based on their physiological behavior during the extended drought stress period (Figure 1). Next, we annotated every dot to the specific vine and variety, and left in the analysis only those varieties in which all four vines are classified into the same group. When looking into the physiological data for the vines represented in each group—the group of varieties marked by green dots—Nitzan 3 (Yael) and Beer, which come from a group that is more separated from the other two groups, are varieties that are characterized by the most negative SWP and lowest stomata conductance at the later stages of the experiment (Figure 2a,b). Meanwhile, the group of varieties marked by red dots—Shami and Batar Nitzamin—had improved parameters (SWP, photosynthesis, and stomata conductance) at the later stages. Finally, the intermediate group marked by the blue dots—Jandaly and Ramtania—showed moderate parameters at the late stages, and had some intermixing with the red group (Figure 2a,b).

The correlation between stomata conductance (g_s_) and photosynthetic efficiency (An) of varieties under drought stress (Figure 2a) shows close to linear relations with high R^2^ levels for most varieties. Nevertheless, the slopes are higher for the stable (group 1) varieties (Batar Nitzanim:24.01, Shami: 23.29) than for those drought-sensitive varieties that show a more extreme reaction (Nitzan: 18.05~18, Beer: 17.92~18), meaning that the red group transpires more water for the same photosynthetic efficiency.

Next, we conducted a correlation between midday stem water potential and stomatal conductance for two representative varieties for each response strategy (four vines each) during all five measuring points (Figure 2b). The correlation clearly shows the differences between the groups—the more stable varieties, Shami and Batar-Nitzanim, are only mildly affected by extended drought, reaching only circa -1MPa, and maintaining relatively high g_s_ at the later stages of the experiment, while the sensitive varieties, Beer and Nitzan 3, are dramatically affected at the early stages, reaching more negative levels of SWP and with lower stomatal conductance. The Ramtania and Jandaly varieties represent the moderate group, dropping faster into a more negative SWP and lower g_s_ than the stable group, but not as dramatically as the sensitive varieties.

### 2.2. Phenological Observations

**Figure 3:**
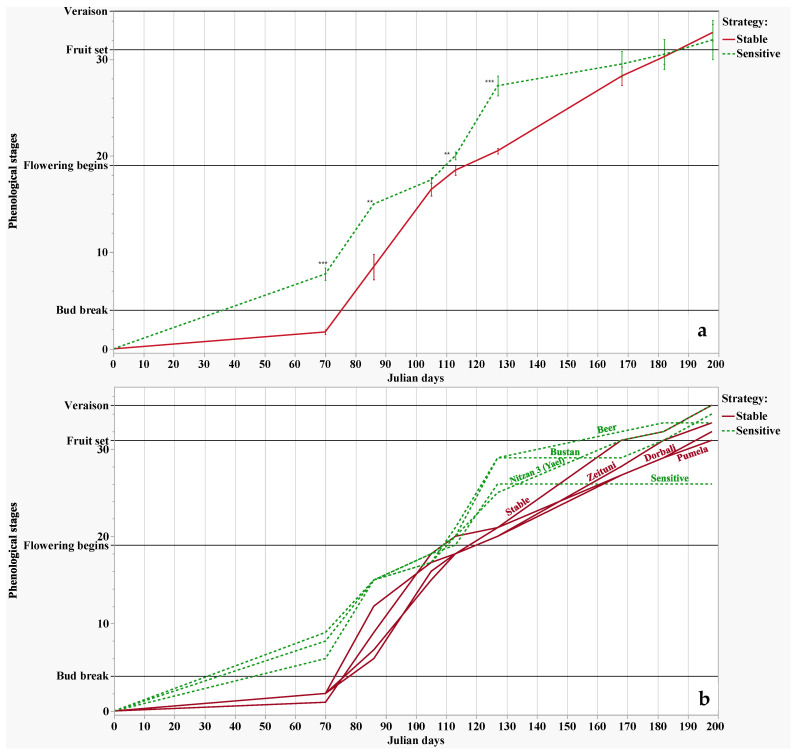
Phenological observations (bud break, flowering, fruit set, and veraison) of grapevine varieties during January to July. (**a**) Average performance for 8 stable species and 7 sensitive species. The statistical analysis we used was the t-test. ** t < 0.01, *** t < 0.001. (**b**) Specific phenological sequence for 4 sensitive (green) and 4 stable (red) varieties showing the highest differences in foliage development.

A comprehensive dating of the phenological stages for the accessions in the collection was conducted during the season of 2018. Determination of the phenological sequence is generally important for the characterization of each variety as an early, medium, or late blooming. When extended to the ripening period, it also enables planning for an extended harvest season for table grapes, and enables planning for early or late harvest for winemaking purposes. Interestingly, when referring to the former division of the varieties by their drought stress reactions, we found that stable varieties show very late bud break and foliage development (Figure 3) compared to sensitive varieties. By fruit set, most varieties show a similar development pattern.

### 2.3. Phenotyping for Various Viticultural Traits

**Figure 4:**
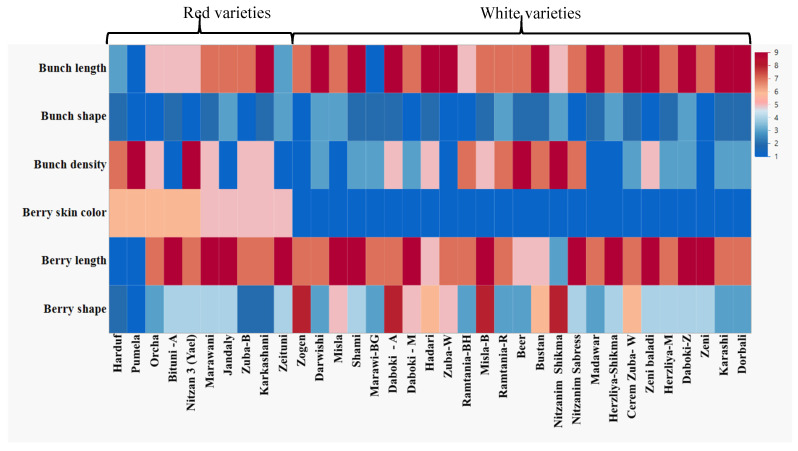
Ampelographical observations (berry shape, berry skin color, berry length, bunch density, bunch shape, bunch length).

When dealing with a collection of newly found varieties that were never before characterized and studied, such as the case for the Israeli indigenous collection, every aspect is essential for describing the newly characterized varieties. Most notable are the fruit characteristics, described by the OIV descriptors [41], which can be presented by heat maps (Figure 4). These characterizations enable the initial categorization of varieties into table or wine grapes, and generally characterize the population to specific geographic groups (proles Orientalis, [42]).

### 2.4. Metabolic Profiling—Phenolics

**Figure 5:**
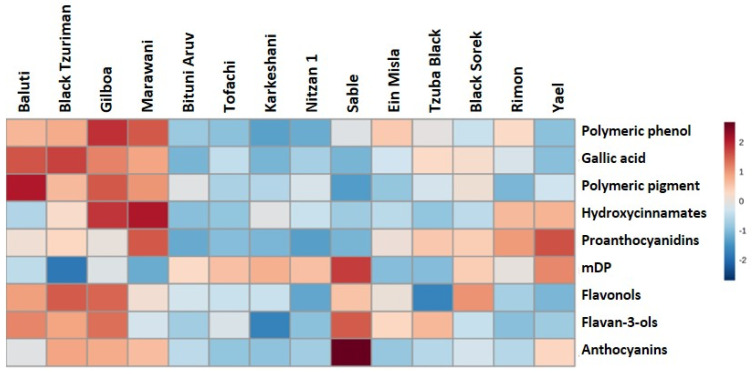
Mean monomeric and proanthocyanidin compositions of indigenous black grape varieties (*n* = 3 replicates per variety). mDP is the mean degree of polymerization, calculated from the sum of all subunits (flavan-3-ol monomer and phloroglucinol adduct, in nmoles, divided by the sum of all flavan-3-ol monomers, in nmoles). All other analytes are reported as mg/g berries in (+) catechin equivalents; for this visualization, mean concentrations were normalized across the observed concentration range, as shown in the figure key. For numerical data, see Supplementary Materials Table S1.

Phenolic profiles of the indigenous black varieties demonstrate a range of monomeric and polymeric phenolic concentrations (Figure 5). Relative to the commercial black table grape cultivar, Sable, the indigenous varieties have lower total anthocyanin concentrations. The indigenous varieties that were analyzed were divided into two major clades; the left clade, including Baluti, Black Tzuriman, Gilboa, and Marawani, show higher polymeric phenols, polymeric pigments, gallic acid, total hydroxycinnamate, flavonol, and flavanol concentrations. These parameters are important in assessing the varieties’ winemaking potential. These samples were collected from the vines under normal conditions with no harsh water stress. However, this example indicates that the availability of in-field collection allows for more detailed comparisons of genetic and climate/environmental interactions on phenolic profiles of these indigenous varieties. It will also provide a unique opportunity to evaluate the impacts of climate change over multiple harvest years, not including the stresses induced by commercial production practices.

### 2.5. Volatile Aroma Profiles

**Figure 6:**
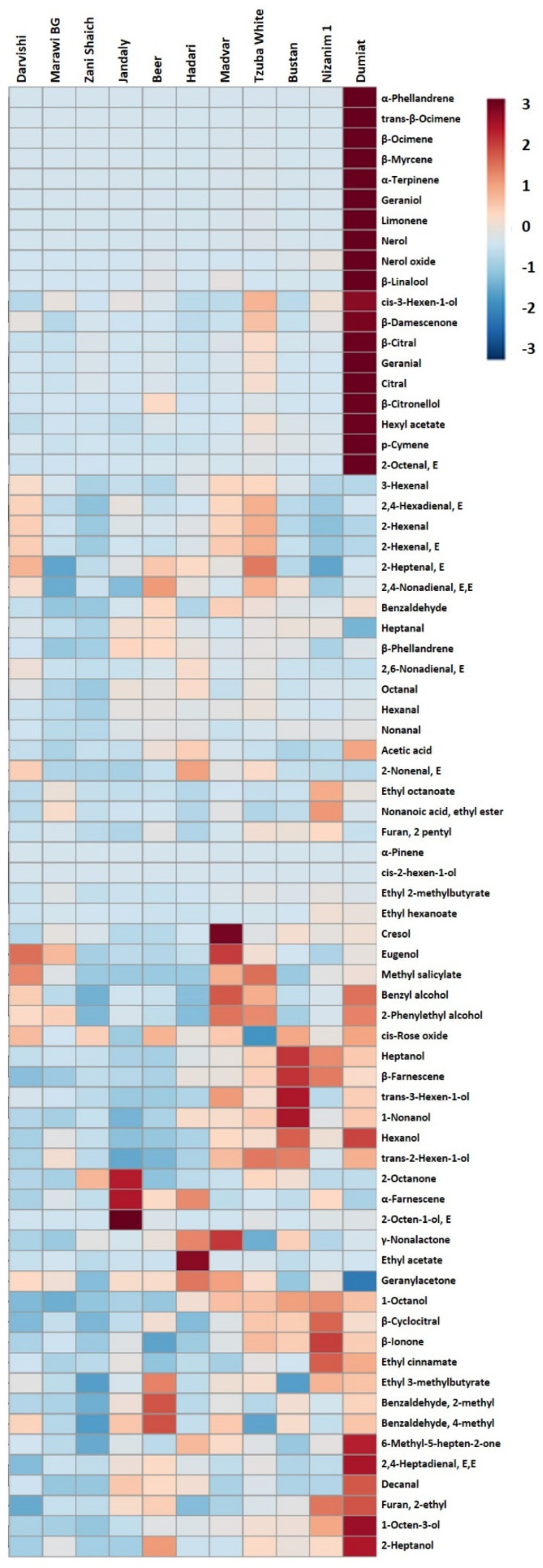
Mean volatile composition of white indigenous cultivars (μg/kg berries; *n* = 3 replicates per variety). For this visualization, mean concentrations were normalized across the observed concentration range, as shown in the figure key. For numerical data, see Supplementary Materials Table S2.

Like the phenolics, the volatile aroma profile of the white indigenous varieties is highly variable (Figure 6). Interestingly, the cultivar Dumiat has a high monoterpene concentration, which was previously unreported. Monoterpenes contribute floral and fruity aromas, and commercial cultivars such as Riesling and Muscat with high monoterpene concentrations are valued for their varietal characters [43,44]. Variations in concentrations of benzene derivatives/phenylpropanoid compounds, such as cresol, eugenol, methyl salicylate, benzyl alcholol, and 2-phenylethyl alcohol, were also observed in these varieties. These compounds can contribute spicy and floral notes to grapes and wines. As a comparison, we included a *V. vinifera* spp. *sylvestris* accession, DVIT3350.06, from the USDA National Clonal Germplasm Repository in Davis, CA USA [45]. This sample has low levels of terpenes and a distinct profile of high to moderate levels of aldehydes, alcohols, and esters, which can contribute green and fruity aromas to the grapes. Overall, in-field germplasms provide a valuable opportunity to obtain information on ‘true’ varietal aroma characteristic for grapes. With this information, new varieties can be identified that provide unique flavor characteristics to meet consumer demands for both table and wine grape production, in a variety of climatic conditions.

## 3. Discussion

Studies of phenotypic variation using germplasm collections can improve our understanding of phenotypic plasticity in grapevine varieties, as they respond to changing climatic conditions. Therefore, it is essential to examine diverse germplasms to better understand variation in individual responses to changing conditions, and to identify germplasms that can withstand climatic variations for use in breeding programs [28,46]. In this paper, we have shown that drought stress, applied uniformly to the whole range of the Israeli germplasm collection which contains six plants for each variety, enables the preliminary identification of drought-stable or drought-sensitive varieties. Stable varieties showed improved SWP and higher levels of stomata conductance under drought stress, while the sensitive varieties dropped in SWP rapidly, as well as in stomata conductance. A group of varieties with a moderated strategy was also characterized. This initial characterization should be followed by a deeper one, concentrating on the varieties that harbor the most pronounced phenotypes in larger dedicated plots and replications. Using a field germplasm collection for this initial screening has pros and cons. Advantageously, we can count the fact that one can conduct comprehensive surveys that encompass the entire range of varieties, to the trait in hand, and the ease of work, when all varieties are under the same conditions in one plot; this facilitates multiple analyses in a short time. In terms of disadvantages, a collection usually contains a very limited number of vines per variety, with no replicate vines spread in different parts of the vineyards, as we would in stationing a proper field experiment. Thus, when analyzing the plasticity of varieties to traits such as stress resistance, the data collected can serve only as preliminary observations to be verified by wider experiments.

Living germplasm collections can also be valuable resources for characterizing diversity in phenological sequences—bloom time, timing of bud break, and véraison, as well as fruit development, ripening processes, and cold hardiness, when responding to identical sets of environmental conditions on the same site across multiple years and conditions [32,33,34,38].

As we have shown, our tracking of the early phenological sequence (bud break, foliage development) in some varieties in our collection is in concert with our findings during the drought stress experiment, and the separation of varieties into “stable” or “sensitive” to drought stress. It seems that the stable varieties are those which are late blooming, while those that are sensitive are early blooming (Figure 3a,b). We suggest that the late bud breaking and foliar development of the “stable” varieties is possibly the mechanism that enables their mild reaction to the initiation of drought stress. The early varieties have around forty additional days to sustain their foliage, possibly causing rapid depletion of the soil water reservoir accumulated during winter. Thus, we suggest that at the point of drought stress initiation (prevention of irrigation, after veraison), their depleted soil water reservoir leads them into more profound stress, preventing them from keeping their stomata open; meanwhile, the late blooming varieties, which have much larger water reservoirs due to less time sustaining foliage, have improved water status. We will need to continue monitoring both phenological and physiological reactions of the stable and sensitive groups over an extended period of time, in order to understand and validate this suggestion; nevertheless, these surprising observations were possible only through maintaining an in-field collection, with meticulous data collection for different traits.

Field germplasm collections can be utilized as reference materials for ampelographic studies [47,48,49,50,51]. Recent research done at the indigenous grapevine collection at Ariel University revealed information about genetic diversity [21,39] and single nucleotide polymorphisms [52] among various accessions, which in combination with the ampelographic data collected in field, can enable the definition of varieties and clones. For example, we have previously shown that seed morphology analyses of three sets of SSR identical pairs of varieties—Shami and Tufahi, Karkashani and Zituni, Baluti and Bituni—found that the first two pairs are clearly distinguishable by seed morphology, while the third pair (Baluti and Bituni) has very similar seed morphology, strengthening this pair’s definition as synonyms [53]. The newly developed method for defining 3D seed morphology joins the more traditional straightforward traits of bunch and berry size and shape, berry color, and taste, which help distinguish synonyms from clones. Indeed, many such variations resulted in the development of popular cultivars such as ‘Pinot gris’ and ‘Pinot blanc’, which are lighter-colored variations of the grapevine cultivar, ‘Pinot noir’, which possesses 17 clones within the USDA grapevine germplasm collection [54,55]. While such accessions may appear to be genotypic duplicates, especially when a limited number of genetic markers is used to evaluate their differences, they are actually valuable resources for studying plant developmental biology and gene function [55].

Here, we deeply analyzed phenolic composition for some red varieties in the collection, and aroma profiles for the white ones. We show that most red indigenous varieties have relatively low anthocyanin and polyphenolics levels. Only four varieties, namely Marawani, Gilboa, Black Tzuriman, and Baluti, showed higher levels of anthocyanins and phenolic characteristics, which may indicate possible suitability for wine production. The Yael variety ael shows moderate levels of anthocyanins, hydroxycinnamates, and proanthocyanidins, as well as a high mean degree of polymerization, but with lower levels of the other measured factors. These compounds can contribute bitter and astringent characteristics to grapes and wines, impacting overall flavor and mouthfeel properties. Wild varieties are an incredibly valuable genetic resource for grape breeding, particularly for enhancing metabolites such as resveratrol [56], as well as for disease resistance [57].

Considering the aroma profiling, most indigenous Israeli varieties showed low levels of terpenoids and esters. Such volatile aroma profiles are often relatively neutral in sensory properties, making wines made from these varieties useful for blending purposes. However, some interesting differences in the volatile profiles were observed that may provide valuable germplasms for breeding unique aroma and flavor traits. For example, a unique high-terpene variety, Dumiat, was observed, which may provide an aromatic quality similar to Muscat and Riesling varieties. In addition, the Madvar variety has a unique profile among this set of varieties that is high in benzenoid/phenylpropanoid compounds such as cresol, eugenol, methyl salicylate, benzyl alcohol, and 2-phenylethanol. Depending on their concentrations, these compounds can contribute spicy, smoky, and honey-like aromas. Further sensory analyses will be necessary to fully relate the chemical composition of these accessions with specific aroma and flavor attributes. However, this initial analysis demonstrates the potential for these field-grown indigenous varieties to serve as sources for breeding new varieties with unique flavor profiles. Such varieties not only provide resources for breeding against a wide range of biotic and abiotic stresses, but also have oenological potential with health benefits [58,59,60].

This high throughput analysis of berries from many different varieties, grown under the same geographic and agronomic conditions, can only be achieved by working in the germplasm collections. This comprehensive analysis can help determine possible uses of these varieties, especially when considering newly found ones that have never been studied and categorized.

## 4. Materials and Methods

### 4.1. In-Field Conditions

The research was carried out at the Israeli indigenous grape variety collection, Ariel, Israel (32.107036, 35.197169). The collection was planted during the years 2013–2015, with additional planting in 2017 (not included in this paper), using plant material collected during our comprehensive survey [39,40]. The plant material was grafted to 140 Ruggeri rootstock, planted, and trained into a double cordon, with vertical shoot positioning. Row direction was north–south with a slight tendency to the west, and vine and row spacing were 1.5 m and 3 m, respectively. The soil is sandy loam. Irrigation is performed regularly using a drip system, with 1 m^3^ water/dunam/day.

### 4.2. Experimental Conditions and Physiological Parameters for the Drought Stress Trial

A drought stress experiment was performed in the “rescue” vineyard from 19 June 2019, to 29 August 2019 (summer season in Israel). Irrigation was performed regularly using a drip system with 1 m^3^ water/dunam/day, until initiation of the drought stress experiment. Pest management and fertilization in the vineyard were applied according to standard local agricultural practices. No rainfall events were recorded during the experimental period, and the average winter rainfall is 520 mm.

The experiment was conducted on thirty-nine indigenous grapevine varieties maintained in the vineyard since 2013 (Figure 1). Prior to experimental research, vines were irrigated for eight hours, once per week for two weeks, in order to achieve water saturation in the rhizosphere. From the third week onwards, these plants were not irrigated for two and half months, in order to maintain drought stress. During the drought stress experiment, midday stem water potential (Ψ_stem_), gas exchange rate via measuring stomatal conductance (g_s_), and carbon fixation/photosynthesis (An), were measured to understand the physiological condition of each variety under drought stress.

#### 4.2.1. Midday Stem Water Potential (Ψ_stem_)

The water potential in the stem was measured for two leaves, leaves that were not too old or not too young (moderately sized fresh leaves, which are exposed to sun and closest to the main trunk/stem of the plant) from each vine. This measurement was made once per week using a pressure chamber (Arimed 3000, MRC Holon), between 12:00–14:00 (midday: warmer period of the day, when minimum variation in leaf temperature is found). For the measurement of midday stem water potential (MD-SWP), one leaf was inserted into a transparent plastic cover, which was further covered with a thin aluminum bag for a minimum 1.5 h prior to measurement of water potential.

#### 4.2.2. Gas Exchange

A portable gas exchange system (LI-6400, Li-Cor, Lincoln, NE, USA) that was fitted with a 6-square-centimeter leaf chamber used to assess the rate of carbon fixation (An) and stomatal conductance (g_s_). All measurements were conducted at a CO_2_ concentration of 400 μmol CO_2_ mol^−1^, air flow rate of 500 μmol air s^−1^, and photosynthetic photon flux density of 1000 μmol m^−2^ s^−1^. Temperature and relative humidity in the chamber were set on the basis of ambient conditions, the measurement hours were at midday 12:00–14:00. Stomata conductance (g_s_) and the rate of photosynthesis (CO_2_ fixation) were measured once every two weeks. The measurements from the intact mature leaf facing towards sun were taken at around solar noon. Mature, fully expanded and healthy leaves were chosen for experimental setups [61].

#### 4.2.3. Statistical Analysis of Physiological Parameters

Each variety had four replicates. K-means cluster statistical analysis was performed (Figure 1; pc is principal components). K-mean cluster was used to reduce the computational load when the number of samples were large, and when the similarity analysis approach of the same variables were in two different groups or more [62,63].

### 4.3. Phenological Stages

In the present study, phenological stages such as bud break, flowering initiation, fruit set, and veraison (berry softening begins; berry coloring begins) were observed according to the guidelines of Coombe [64]. Phenological parameters were observed for each variety, with a minimum of six replicates.

### 4.4. Ampelographic Characterization of Grapevine Varieties

The classification of grapevine varieties and their morphological characterization were carried out using six categories, such as berry (shape, length, and skin color) and bunch (length, shape, and density), according to Organisation Internationale de la Vigne et du Vin (OIV) [48].

### 4.5. Volatile Aroma Compounds

Volatile aroma compounds were profiled using the basic headspace solid-phase microextraction (HS-SPME) gas chromatography mass spectrometry (GC-MS) methods of Hendrickson et al. (2016) [65] and Canuti et al. (2009) [66]. For each sample, grape powder was obtained from slices of whole berries that were harvested at 20 Brix in 2019, which were homogenized in liquid nitrogen using an Ultra-Turax T 18 basic disperser (IKA Works, Inc., Guangzhou, China) and stored at −80 °C until further use. At the time of analysis, 1 g of frozen powder was accurately weighed in a 10-milliliter amber headspace vial containing 0.5 g of NaCl, 0.5 mL of sodium citrate buffer (1.0 M, pH 6.0), 20 uL of a 10 mg/L 2-octannol internal standard solution (prepared in 100% ethanol), and 20 uL of 2-undecanone solution (10 mg/L in 100% ethanol) as a second internal standard. The homogenate was mixed properly, and the headspace vials containing the grape homogenates were sealed with magnetic screwcaps with PTFE septa for HS-SPME GC MS analysis. Prior to HS-SPME sampling, samples were equilibrated in the GC autosampler at 30 °C for 5 min, with agitation at 500 rpm.

Prior to HS-SPME sampling, samples were equilibrated in a GC autosampler at 30 °C for 5 min, with agitation at 500 rpm. The SPME fiber (1 cm PDMS, 23 gauge, Supelco, Bellefonte, PA, USA) was then inserted into the sample vial, and the headspace was extracted for 45 min at 30 °C, with agitation at 250 rpm. After extraction, the SPME fiber was inserted into the GC inlet at 260 °C (0.7-mm i.d. inlet liner, Supelco), and the sample was desorbed in splitless mode; the split vent subsequently was opened at 1.2 min. The electron impact source had a 2-min solvent delay, and the detector was turned off from 3.80 min to 4.30 min during ethanol elution. 

A 6890 gas chromatograph (Agilent, Inc., DE, USA) with a 5975 mass selective detector (MSD, Agilent) and MPS2 autosampler (Gerstel, Inc., Linthicum, MD, USA), controlled by Maestro (ver. 1.2.3.1, Gerstel) software, was used for volatiles analysis. Separation was performed on a DB-Wax ETR capillary column (30 m, 0.25-mm i.d., 0.25-micrometer film thickness; J&W Scientific, Folsom, CA, USA), with helium carrier gas (constant pressure, 6.69 psi) and a retention time locking to 2-undecanone, at constant pressure to prevent retention time drifting. The GC oven was programmed with an initial hold at 40 °C for 5 min, followed by a first temperature ramp at 3 °C/min to 180 °C, and a second ramp at 30 °C/min to 260 °C, with a final hold at 260 °C for 7.67 min. The MSD interface was held at 260 °C, the electron impact source temperature was 230 °C, and the quadrupole was held at 150 °C. Samples were analyzed in synchronous scan and selected ion monitoring mode. The scan range used was from 40 *m/z* to 300 *m/z*, and compounds were detected using between two and six selected ions with a scan rate of 5.8 scans/sec, as described in Hendrickson et al. (2016) [65].

The relative concentrations (μg/kg berries) of 72 compounds were determined by normalizing peak areas to both internal standards and compounds that were identified based on a comparison of retention times and spectra to authentic standards [65]. All samples were analyzed in triplicate. Means and standard errors were calculated in Excel.

### 4.6. Phenolics

The phenolic composition was determined for a subset of 14 black indigenous varieties, harvested at 20 Brix in 2019. Sable, a commercial black table grape cultivar, was also included as a reference. Monomeric phenolics in homogenates of 0.8-milligram frozen grape powder (described above) were analyzed using reverse phase HPLC with diode array detection, and proanthocyanidins were analyzed via phloroglucinolysis, according to the methods described in Tyagi et al. (2022) [67], without modification.

## 5. Conclusions

Living *Vitis* germplasm collections can be a valuable resource for the characterization of grape varieties, for the selection of new wine and table varieties with unique properties, and for the identification of varieties that are resistant to stress—plasticity is critical in the face of climate change We demonstrated that some insights can only be achieved when crossing data from various measurements in the same collection.

## Data Availability

Not applicable.

## Supplementary Materials

The following supporting information can be downloaded at: https://www.mdpi.com/2223-7747/11/19/2563/s1, Table S1: Monomeric and proanthocyanidin composition of indigenous black grape varieties numerical data; Table S2: Volatile composition of white indigenous cultivars numerical data.

## References

[1] Imazio S., Maghradze D., de Lorenzis G., Bacilieri R., Laucou V., This P., Scienza A., Failla O. (2013). From the Cradle of Grapevine Domestication: Molecular Overview and Description of Georgian Grapevine (*Vitis vinifera* L.) Germplasm. Tree Genet. Genomes.

[2] Santiago J.L., Boso S., Gago P., Alonso-Villaverde V., Martínez M.C. (2008). A Contribution to the Maintenance of Grapevine Diversity: The Rescue of Tinta Castañal (*Vitis vinifera* L.), a Variety on the Edge of Extinction. Sci. Hortic..

[3] Maraš V., Tello J., Gazivoda A., Mugoša M., Perišić M., Raičević J., Štajner N., Ocete R., Božović V., Popović T. (2020). Population Genetic Analysis in Old Montenegrin Vineyards Reveals Ancient Ways Currently Active to Generate Diversity in *Vitis vinifera*. Sci. Rep..

[4] Harst-Langenbucher M., Alleweldt G. (1990). Conservation of the Genetic Resources of *Vitis*. Vitis.

[5] Kulus D., Zalewska M. (2014). Cryopreservation as a Tool Used in Long-Term Storage of Ornamental Species—A Review. Sci. Hortic..

[6] Bhojwani S.S., Razdan M.K., Bhojwani S.S., Razdan M.K. (1996). Plant Tissue Culture Theory and Practice, a Revised Edition.

[7] Bi W.-L., Pan C., Hao X.-Y., Cui Z.-H., Kher M.M., Marković Z., Wang Q.-C., Teixeira da Silva J.A. (2017). Cryopreservation of Grapevine (*Vitis* Spp.)—A Review. Vitr. Cell. Dev. Biol.—Plant.

[8] Bettoni J.C., Marković Z., Bi W., Volk G.M., Matsumoto T., Wang Q. (2021). Grapevine Shoot Tip Cryopreservation and Cryotherapy: Secure Storage of Disease-Free Plants. Plants.

[9] Karlsson J.O.M., Toner M. (1996). Long-Term Storage of Tissues by Cryopreservation: Critical Issues. Biomaterials.

[10] Carra A., Carimi F., Bettoni J.C., Pathirana R., Faisal M., Alatar A. (2019). Progress and Challenges in the Application of Synthetic Seed Technology for Ex situ Germplasm Conservation in Grapevine (*Vitis* spp.). Synthetic Seeds.

[11] Butler D. (2014). Grapevine Gene Bank under Threat. Nature.

[12] Meng B., Martelli G.P., Golino D.A., Fuchs M., Meng B., Martelli G.P., Golino D.A., Fuchs M. (2017). Grapevine Viruses: Molecular Biology, Diagnostics and Management.

[13] Burr T.J., Otten L. (1999). Crown Gall of Grape: Biology and Disease Management. Annu. Rev. Phytopathol..

[14] Molitor D., Beyer M. (2014). Epidemiology, Identification and Disease Management of Grape Black Rot and Potentially Useful Metabolites of Black Rot Pathogens for Industrial Applications—A Review. Ann. Appl. Biol..

[15] Boso S., Alonso-Villaverde V., Gago P., Santiago J.L., Martínez M.C. (2014). Susceptibility to Downy Mildew (*Plasmopara viticola*) of Different *Vitis* Varieties. Crop Prot..

[16] Li Z., dos Santos R.F., Gao L., Chang P., Wang X. (2021). Current Status and Future Prospects of Grapevine Anthracnose Caused by *Elsinoe ampelina*: An Important Disease in Humid Grape-Growing Regions. Mol. Plant Pathol..

[17] Pettenuzzo S., Cappellin L., Grando M.S., Costantini L. (2022). Phenotyping Methods to Assess Heat Stress Resilience in Grapevine. J. Exp. Bot..

[18] Gambetta G.A., Herrera J.C., Dayer S., Feng Q., Hochberg U., Castellarin S.D. (2020). The Physiology of Drought Stress in Grapevine: Towards an Integrative Definition of Drought Tolerance. J. Exp. Bot..

[19] Miras-Avalos J.M., Araujo E.S. (2021). Optimization of Vineyard Water Management: Challenges, Strategies, and Perspectives. Water.

[20] Droulia F., Charalampopoulos I. (2021). Future Climate Change Impacts on European Viticulture: A Review on Recent Scientific Advances. Atmosphere.

[21] Sivan A., Rahimi O., Lavi B., Salmon-Divon M., Weiss E., Drori E., Hübner S. (2021). Genomic Evidence Supports an Independent History of Levantine and Eurasian Grapevines. Plants People Planet.

[22] Klein L.L., Miller A.J., Ciotir C., Hyma K., Uribe-Convers S., Londo J. (2018). High-Throughput Sequencing Data Clarify Evolutionary Relationships among North American *Vitis* Species and Improve Identification in USDA Vitis Germplasm Collections. Am. J. Bot..

[23] Cretazzo E., Moreno Sanz P., Lorenzi S., Benítez M.L., Velasco L., Emanuelli F. (2022). Genetic Characterization by SSR Markers of a Comprehensive Wine Grape Collection Conserved at Rancho de La Merced (Andalusia, Spain). Plants.

[24] Community of Madrid Collection of Vine Varieties “Grapevine Collection of “El Encín”. https://www.comunidad.madrid/servicios/medio-rural/coleccion-variedades-vid.

[25] Emanuelli F., Lorenzi S., Grzeskowiak L., Catalano V., Stefanini M., Troggio M., Myles S., Martinez-Zapater J.M., Zyprian E., Moreira F.M. (2013). Genetic Diversity and Population Structure Assessed by SSR and SNP Markers in a Large Germplasm Collection of Grape. BMC Plant Biol..

[26] VIVC Vitis International Variety Catalogue. https://www.vivc.de/index.php?r=cultivarname%2Findex.

[27] Lubin B.C.R., Inbar N., Pinkus A., Stanevsky M., Cohen J., Rahimi O., Anker Y., Shoseyov O., Drori E. (2022). Ecogeographic Conditions Dramatically Affect Trans-Resveratrol and Other Major Phenolics’ Levels in Wine at a Semi-Arid Area. Plants.

[28] Wolkovich E.M., García De Cortázar-Atauri I., Morales-Castilla I., Nicholas K.A., Lacombe T. (2018). From Pinot to Xinomavro in the World’s Future Wine-Growing Regions. Nat. Clim. Chang..

[29] Jones G.V., E D.R. (2000). Climate Influences on Grapevine Phenology, Grape Composition, and Wine Production and Quality for Bordeaux, France. Am. J. Enol. Vitic..

[30] Gutiérrez-Gamboa G., Liu S.Y., Pszczólkowski P. (2020). Resurgence of Minority and Autochthonous Grapevine Varieties in South America: A Review of Their Oenological Potential. J. Sci. Food Agric..

[31] Migicovsky Z., Warschefsky E., Klein L.L., Miller A.J. (2019). Using Living Germplasm Collections to Characterize, Improve, and Conserve Woody Perennials. Crop Sci..

[32] Kovaleski A.P., Reisch B.I., Londo J.P. (2018). Deacclimation Kinetics as a Quantitative Phenotype for Delineating the Dormancy Transition and Thermal Efficiency for Budbreak in *Vitis* Species. AoB PLANTS.

[33] Londo J.P., Kovaleski A.P. (2019). Deconstructing Cold Hardiness: Variation in Supercooling Ability and Chilling Requirements in the Wild Grapevine *Vitis riparia*. Aust. J. Grape Wine Res..

[34] Gottschalk C., Van Nocker S. (2013). Diversity in Seasonal Bloom Time and Floral Development among Apple Species and Hybrids. J. Am. Soc. Hortic. Sci..

[35] Drori E., Munitz S., Pinkus A., Stanevsky M., Netzer Y. (2022). The Effect of Irrigation-Initiation Timing on the Phenolic Composition and Overall Quality of Cabernet Sauvignon Wines Grown in a Semi-Arid Climate. Foods.

[36] Sorek Y., Greenstein S., Netzer Y., Shtein I., Jansen S., Hochberg U. (2021). An Increase in Xylem Embolism Resistance of Grapevine Leaves during the Growing Season Is Coordinated with Stomatal Regulation, Turgor Loss Point and Intervessel Pit Membranes. New Phytol..

[37] Zhou-Tsang A., Wu Y., Henderson S.W., Walker A.R., Borneman A.R., Walker R.R., Gilliham M. (2021). Grapevine Salt Tolerance. Aust. J. Grape Wine Res..

[38] Venios X., Korkas E., Nisiotou A., Banilas G. (2020). Grapevine Responses to Heat Stress and Global Warming. Plants.

[39] Drori E., Rahimi O., Marrano A., Henig Y., Brauner H., Salmon-Divon M., Netzer Y., Prazzoli M.L., Stanevsky M., Failla O. (2017). Collection and Characterization of Grapevine Genetic Resources (*Vitis vinifera*) in the Holy Land, towards the Renewal of Ancient Winemaking Practices. Sci. Rep..

[40] Drori E., Rahimi O., Henig Y., Lorenzi S., Brauner H., Marrano A., Amar Z., Netzer Y., Failla O., Grando M.S. (2015). Ampelographic and Genetic Characterization of an Initial Israeli Grapevine Germplasm Collection. Vitis—J. Grapevine Res..

[41] Muñoz G., Gaforio L., Muñoz S., Cabello F. (2011). Manual for Standadization of OIV Vitis Descriptors.

[42] Negrul A.M., Baranov A., Kai Y.F., Lazarevski M.A., Palibin T.V., Prosmoserdov N.N. (1946). Origin of Cultivated Grapevine and Its Classification. Ampelography of the Soviet Union.

[43] Mateo J.., Jiménez M. (2000). Monoterpenes in Grape Juice and Wines. J. Chromatogr. A.

[44] Marais J. (1983). Terpenes in the Aroma of Grapes and Wines: A Review. S. Afr. J. Enol. Vitic..

[45] Riaz S., De Lorenzis G., Velasco D., Koehmstedt A., Maghradze D., Bobokashvili Z., Musayev M., Zdunic G., Laucou V., Andrew Walker M. (2018). Genetic Diversity Analysis of Cultivated and Wild Grapevine (*Vitis vinifera* L.) Accessions around the Mediterranean Basin and Central Asia. BMC Plant Biol..

[46] Sargolzaei M., Rustioni L., Cola G., Ricciardi V., Bianco P.A., Maghradze D., Failla O., Quaglino F., Toffolatti S.L., De Lorenzis G. (2021). Georgian Grapevine Cultivars: Ancient Biodiversity for Future Viticulture. Front. Plant Sci..

[47] Vršič S. (2012). An Overwiew of Ampelographic Research and Modifications of Grapevine Assortment. Agricultura.

[48] Organisation Internationale de la Vigne et du Vin (OIV) Second Edition of the OIV Descriptor List for Grape Varieties and Vitis Species. https://www.oiv.int/public/medias/2274/code-2e-edition-finale.pdf.

[49] Maletić E., Pejić I., Karoglan Kontić J., Zdunić G., Preiner D., Šimon S., Andabaka Ž., Žuljmihaljević M., Bubola M., Marković Z. (2015). Ampelographic and Genetic Characterization of Croatian Grapevine Varieties. Vitis—J. Grapevine Res..

[50] Rustioni L., Cola G., Maghradze D., Abashidze E., Argiriou A., Aroutiounian R., Brazão J., Chipashvili R., Cocco M., Cornea V. (2019). Description of the *Vitis vinifera* L. Phenotypic Variability in Eno-Carpological Traits by a Euro-Asiatic Collaborative Network among Ampelographic Collections. Vitis—J. Grapevine Res..

[51] Rustioni L., Maghradze D., Popescu C.F., Cola G., Abashidze E., Aroutiounian R., Brazão J., Coletti S., Cornea V., Dejeu L. (2014). First Results of the European Grapevine Collections’ Collaborative Network: Validation of a Standard Eno-Carpological Phenotyping Method. Vitis—J. Grapevine Res..

[52] Drori E., Levy D., Smirin-Yosef P., Rahimi O., Salmon-Divon M. (2017). CircosVCF: Circos Visualization of Whole-Genome Sequence Variations Stored in VCF Files. Bioinformatics.

[53] Karasik A., Rahimi O., David M., Weiss E., Drori E. (2018). Development of a 3D Seed Morphological Tool for Grapevine Variety Identification, and Its Comparison with SSR Analysis. Sci. Rep..

[54] Myles S., Boyko A.R., Owens C.L., Brown P.J., Grassi F., Aradhya M.K., Prins B., Reynolds A., Chia J.M., Ware D. (2011). Genetic Structure and Domestication History of the Grape. Proc. Natl. Acad. Sci. USA.

[55] Foster T.M., Aranzana M.J. (2018). Attention Sports Fans! The Far-Reaching Contributions of Bud Sport Mutants to Horticulture and Plant Biology. Hortic. Res..

[56] Li R., Xie X., Ma F., Wang D., Wang L., Zhang J., Xu Y., Wang X., Zhang C., Wang Y. (2017). Resveratrol Accumulation and Its Involvement in Stilbene Synthetic Pathway of Chinese Wild Grapes during Berry Development Using Quantitative Proteome Analysis. Sci. Rep..

[57] Hu Y., Cheng Y., Yu X., Liu J., Yang L., Gao Y., Ke G., Zhou M., Mu B., Xiao S. (2021). Overexpression of Two CDPKs from Wild Chinese Grapevine Enhances Powdery Mildew Resistance in *Vitis vinifera* and *Arabidopsis*. New Phytol..

[58] Gutiérrez-Gamboa G., Liu S.Y., Sun X.Y., Fang Y. (2020). Oenological Potential and Health Benefits of Chinese Non-*Vitis vinifera* Species: An Opportunity to the Revalorization and to Breed New Varieties. Food Res. Int..

[59] Lin J., Massonnet M., Cantu D. (2019). The Genetic Basis of Grape and Wine Aroma. Hortic. Res..

[60] Ju Y.L., Yue X.F., Cao X.Y., Wei X.F., Fang Y.L. (2021). First Study on the Fatty Acids and Their Derived Volatile Profiles from Six Chinese Wild Spine Grape Clones (*Vitis davidii* Foex). Sci. Hortic..

[61] Shtein I., Wolberg S., Munitz S., Zait Y., Rosenzweig T., Grünzweig J.M., Ohana-Levi N., Netzer Y. (2021). Multi-Seasonal Water-Stress Memory versus Temperature-Driven Dynamic Structural Changes in Grapevine. Tree Physiol..

[62] Bock H.-H., Brito P., Cucumel G., Bertrand P., de Carvalho F. (2007). Clustering Methods: A History of k-Means Algorithms. Selected Contributions in Data Analysis and Classification. Studies in Classification, Data Analysis, and Knowledge Organization.

[63] Ahmed M., Seraj R., Islam S.M.S. (2020). The K-Means Algorithm: A Comprehensive Survey and Performance Evaluation. Electronics.

[64] Coombe B.G. (1995). Growth Stages of the Grapevine: Adoption of a System for Identifying Grapevine Growth Stages. Aust. J. Grape Wine Res..

[65] Hendrickson D.A., Lerno L.A., Hjelmeland A.K., Ebeler S.E., Heymann H., Hopfer H., Block K.L., Brenneman C.A., Oberholster A. (2016). Impact of Mechanical Harvesting and Optical Berry Sorting on Grape and Wine Composition. Am. J. Enol. Vitic..

[66] Canuti V., Conversano M., Calzi M.L., Heymann H., Matthews M.A., Ebeler S.E. (2009). Headspace Solid-Phase Microextraction–Gas Chromatography–Mass Spectrometry for Profiling Free Volatile Compounds in Cabernet Sauvignon Grapes and Wines. J. Chromatogr. A.

[67] Tyagi K., Lerno L., De Rosso M., Maoz I., Lichter A., Ebeler S.E., Flamini R., Fett-Neto A.G. (2022). Extraction and Analysis of Phenolic Compounds from Grape Berries. Plant Secondary Metabolism Engineering. Methods in Molecular Biology, Vol. 2469.

