# Supplementary material for: A Field Collection of Indigenous Grapevines as a Valuable Repository for Applied Research
Source: Plants (Basel). 2022 Sep 28;11(19):2563. doi: 10.3390/plants11192563 (PMC9570891; doi:10.3390/plants11192563)
Supplement: Supplementary file 1 [file plants-11-02563-s001.zip › Supplementry table for Fig 5 Phenolics individual.pdf]

Supplementary Table S1: Phenolics monomers and proanthocyanidin composition of indigenous black grape varieties. All values are in mg g^{-1} FW (n = 3). Abbreviations are EGC – epigallocatechin; EC – epicatechin; ECG - epicatechin gallate; quer – quercetin; delph-3-gluc - delphinidin-3-*O*-glucoside; cya-3-gluc - cyanidin-3-*O*-glucoside; pet-3-gluc - petunidin-3-*O*-glucoside; peo-3-gluc - peonidin-3-*O*-glucoside; mal-3-gluc - malvidin-3-*O*-glucoside; delph-3-acetlygluc - delphinidin-3-*O*-acetylglucoside; pet-3-acetlygluc - petunidin-3-*O*-acetylglucoside; peo-3-acetlygluc - peonidin-3-*O*-acetylglucoside; mal-3-acetlygluc - malvidin-3-*O*-acetylglucoside; delph-3-*p*-coumgluc - delphinidin-3-*O*-(6-*p*-coumaroyl) glucoside; pet-3-*p*-coumgluc - petunidin-3-*O*-(6-*p*-coumaroyl) glucoside; peo-3-*p*-coumgluc - peonidin-3-*O*-(6-*p*-coumaroyl) glucoside; mal-3-*p*-coumgluc - malvidin-3-*O*-(6-*p*-coumaroyl) glucoside.

Compounds	Yael	Rimon	Black Sorek	Black Tzuriman	Gilboa	Baluti	Tofachi	Nitzan 1	Ein Misla	Tzuba Black	Marawani	Bituni Aruv	Karkeshani	Sable
**Gallic acid**	0.0001±0	0.0006±0	0.0009±0	0.0019±0	0.0015±0	0.0018±0.001	0.0004±0	0.0003±0	0.0005±0	0.0009±0	0.0013±0.001	0±0	0±0	0±0
**EGC**	0.0153±0.002	0.0106±0.001	0.0254±0	0.0075±0.001	0.0232±0.001	0.0425±0.002	0.0083±0.001	0.0087±0.001	0.0099±0	0.0121±0	0.0211±0.002	0.0205±0	0.008±0	0.031±0.002
**Catechin**	0.0096±0	0.0152±0.002	0.0127±0.002	0.0503±0.01	0.0344±0.006	0.02±0.001	0.0162±0.001	0.0101±0.001	0.0363±0.008	0.0222±0.002	0.024±0.001	0.0149±0.001	0.0068±0.001	0.009±0.001
**EC**	0.0057±0.001	0.0044±0.001	0.0027±0.001	0.0023±0.001	0.0215±0.001	0.0136±0.001	0.0209±0	0.013±0.001	0.0115±0.001	0.0304±0.003	0±0	0±0	0±0	0.044±0.001
**ECG**	0.0045±0.002	0.0006±0	0.0024±0	0.0107±0.005	0.0016±0	0.0007±0	0.0021±0	0±0	0.0011±0.001	0.0015±0	0.0012±0	0±0	0±0	0±0
**Polymeric phenol**	0.5353±0.037	1.0016±0.043	0.7532±0.003	1.1987±0.116	1.6095±0.075	1.1594±0.06	0.5347±0.004	0.4417±0.025	1.0846±0.202	0.8697±0.028	1.4821±0.027	0.5868±0.054	0.3908±0.006	0.85±0.051
**Caftaic acid**	0.1594±0.002	0.1487±0.004	0.0769±0.003	0.1207±0.003	0.2341±0.002	0.0657±0.001	0.0475±0.002	0.0856±0.003	0.0744±0.002	0.0485±0.001	0.2218±0.004	0.044±0.002	0.1086±0.002	0.061±0.001
**Coutaric acid**	0.0177±0	0.0239±0.002	0.0086±0	0.0204±0.001	0.0275±0.001	0.0121±0	0.0069±0	0.0089±0.001	0.0094±0	0.0058±0	0.0603±0.002	0.0062±0	0.0061±0	0.003±0
**Myrcetin glycosides**	0.0634±0.002	0.0108±0	0.1729±0.011	0.1789±0.011	0.161±0.011	0.1652±0.017	0.0204±0.002	0.0625±0.006	0.1257±0.006	0.0367±0.006	0.1513±0.012	0.0984±0.004	0.1106±0.013	0.207±0.012
**Quer-galact**	0.0187±0.003	0.0096±0.002	0.0036±0	0.0092±0	0.0044±0	0.0035±0.001	0.0114±0.003	0.0016±0	0.003±0	0.0008±0	0.0034±0.001	0.0035±0.001	0.0033±0	0.004±0
**Quer-gluco**	0.0264±0.001	0.0693±0.011	0.0224±0.001	0.0698±0.003	0.02±0	0.0207±0.004	0.0914±0.02	0.0135±0.001	0.0197±0.001	0.0061±0.001	0.0172±0.005	0.0197±0.003	0.0219±0.003	0.009±0.0004
**Quer-glucuro**	0±0	0.0375±0.007	0.0538±0.003	0.027±0.001	0.0789±0.002	0.046±0.007	0.0282±0.003	0.0143±0	0.0372±0.001	0.0157±0.001	0.0322±0.002	0.0348±0.002	0.0234±0.002	0±0
**Quer-rhamno**	0.0098±0.001	0.0193±0.003	0.0285±0.001	0.0335±0.001	0.0481±0.002	0.0369±0.004	0.02±0.002	0.0117±0.001	0.0194±0.001	0.0098±0.001	0.0066±0	0.0237±0.001	0.0122±0.001	0.024±0.001
**Delp-3-glu**	0.1292±0.001	0.0091±0	0.0632±0.002	0.2342±0.01	0.0877±0.005	0.0504±0.002	0.0117±0.001	0.0254±0.001	0.0345±0.011	0.0352±0.002	0.1124±0.006	0.0305±0.001	0.0251±0.001	0.338±0.014
**Cya-3-glu**	0.016±0	0.0864±0.004	0.0103±0	0.0847±0.003	0.0027±0	0.005±0	0.0448±0.007	0.0058±0	0.0033±0.001	0.009±0	0.0513±0.004	0.002±0	0.0018±0	0.089±0
**Pet-3-glu**	0.1451±0.001	0.0093±0	0.0789±0.002	0.2429±0.01	0.1113±0.005	0.0635±0.003	0.0126±0.001	0.0288±0.002	0.0406±0.013	0.0483±0.002	0.1726±0.007	0.0402±0.002	0.0269±0.001	0.35±0.014
**Peo-3-glu**	0.1585±0.002	0.8871±0.044	0.1013±0.002	0.7967±0.009	0.0617±0.003	0.054±0.003	0.3666±0.015	0.0693±0.001	0.0289±0.009	0.1103±0.005	0.2347±0.017	0.025±0.002	0.0159±0	0.844±0.013
**Mal-3-glu**	1.4128±0.012	0.1009±0.009	0.9453±0.006	1.4092±0.051	1.77±0.013	0.9652±0.036	0.0999±0.009	0.3881±0.024	0.3473±0.112	0.5815±0.007	1.2583±0.028	0.5761±0.028	0.2346±0.004	2.97±0.089
**Del-3-acetylglu**	0.0048±0	0±0	0.002±0	0.0045±0	0.0079±0	0.0021±0.001	0±0	0.0016±0	0.0018±0.001	0.0018±0	0.0126±0	0.0003±0	0.0016±0	0.024±0.001
**Cya-3-acetylglu**	0±0	0.0024±0	0.0021±0.001	0.0025±0	0.0078±0.001	0.0028±0	0.0013±0	0.0016±0	0.0009±0	0.0019±0	0.0035±0	0.0017±0	0.0001±0	0±0

**Pet-3-acetylglu**	0.0065±0	0.002±0.001	0.0025±0	0.0071±0	0.0123±0	0.002±0	0±0	0.0017±0	0.0021±0.001	0.0021±0	0.0258±0	0.0018±0	0.0024±0	0±0
**Peo-3-acetylglu**	0.011±0	0±0	0±0	0±0	0±0	0±0	0±0	0±0	0±0	0±0	0±0	0±0	0±0	0±0
**Mal-3-acetylglu**	0.1214±0.001	0.0089±0.006	0.0714±0.002	0.0689±0.002	0.4254±0.014	0.0848±0.003	0.0023±0	0.0402±0.002	0.0228±0.007	0.0485±0.001	0.2686±0.003	0.0534±0.003	0.0446±0.001	0.479±0.013
**Delp-3-p-coumgluc**	0.0324±0	0±0	0.0136±0	0.0518±0.002	0.0273±0	0.0164±0	0.0016±0	0.0063±0.001	0.0075±0.002	0.009±0	0.0613±0.001	0.0129±0.001	0.009±0	0.079±0.002
**Cya-3-p-coumgluc**	0±0	0±0	0±0	0±0	0.0108±0	0±0	0.0028±0	0±0	0.001±0.001	0.0029±0	0.0267±0.002	0.0021±0	0.0007±0	0±0
**Pet-3-p-coumgluc**	0.0625±0.001	0.0024±0	0.0375±0.001	0.0662±0.002	0.0682±0.001	0.0568±0.001	0.0007±0.001	0.0176±0.001	0.0182±0.006	0.0257±0	0.107±0.002	0.045±0.002	0.0207±0.001	0±0
**Peo-3-p-coumgluc**	0.0624±0.001	0.0243±0.001	0.029±0.001	0.1505±0.003	0.0214±0	0.0249±0.001	0.0208±0	0.0236±0.001	0.0086±0.003	0.0366±0.002	0.1098±0.006	0.0132±0.001	0.0052±0	0.319±0.005
**Mal-3-p-coumgluc**	0.541±0.001	0.0171±0.002	0.335±0.01	0.4896±0.028	0.8929±0.036	0.6246±0.011	0.0182±0.002	0.209±0.013	0.1214±0.037	0.2373±0.006	0.7362±0.01	0.4676±0.029	0.1621±0.002	1.187±0.02
**Polymeric pigment**	0.0583±0.006	0.0238±0.002	0.0765±0.009	0.1033±0.007	0.1434±0.021	0.1682±0.011	0.0424±0.004	0.0634±0.005	0.0328±0	0.061±0.002	0.1195±0.009	0.0678±0.006	0.0457±0	0.159±0.002
**mDP**	24.63±1	21.13±2.23	22.5±1.04	15.64±1.04	20.77±0.16	19.71±0.23	22.95±0.26	22.99±1.08	18.25±0.37	18.14±0.16	17.55±0.7	22.02±1.13	23.44±0.26	26.5 ± 0.7
**Tannin**	1.94±0.04	1.61±0.02	1.33±0.04	1.26±0.12	1.1±0.05	1.15±0.04	0.57±0.02	0.38±0.01	1.13±0.06	1.36±0.06	1.91±0.07	0.45±0.01	0.52±0.04	0.51 ± 0.06

mDP = mean degree of polymerization, calculated by the sum of all subunits (flavan-3-ol monomer and phloroglucinol adduct, in nmoles), divided by the sum of all flavan-3-ol monomers (in nmoles).
